# Structural and Functional Features of the P2X4 Receptor: An Immunological Perspective

**DOI:** 10.3389/fimmu.2021.645834

**Published:** 2021-03-25

**Authors:** Jean M. Kanellopoulos, Cássio Luiz Coutinho Almeida-da-Silva, Sirje Rüütel Boudinot, David M. Ojcius

**Affiliations:** ^1^Institute for Integrative Biology of the Cell (I2BC), CEA, CNRS, Université Paris-Saclay, Gif-sur-Yvette, France; ^2^Department of Biomedical Sciences, University of the Pacific, Arthur A. Dugoni School of Dentistry, San Francisco, CA, United States; ^3^Department of Chemistry and Biotechnology, Tallinn University of Technology, Tallinn, Estonia

**Keywords:** P2X4 receptor, P2X7 receptor, purinergic receptor, innate immunity, inflammasome, NLRP3, ATP, anti-P2X4 mAb

## Abstract

Extracellular nucleotides are important mediators of activation, triggering various responses through plasma membrane P2 and P1 receptors. P2 receptors are further subdivided into ionotropic P2X receptors and G protein-coupled P2Y receptors. P2X4 is an ATP-gated cation channel broadly expressed in most tissues of the body. Within the P2X family, P2X4 has a unique subcellular distribution, being preferentially localized in lysosomes. In these organelles, high ATP concentrations do not trigger P2X4 because of the low pH. However, when the pH increases to 7.4, P2X4 can be stimulated by intra-lysosomal ATP, which is in its active, tetra-anionic form. Elucidation of P2X4, P2X3 and P2X7 structures has shed some light on the functional differences between these purinergic receptors. The potential interaction between P2X4 and P2X7 has been extensively studied. Despite intensive effort, it has not been possible yet to determine whether P2X4 and P2X7 interact as heterotrimers or homotrimers at the plasma membrane. However, several publications have shown that functional interactions between P2X4 and P2X7 do occur. Importantly, these studies indicate that P2X4 potentiates P2X7-dependent activation of inflammasomes, leading to increased release of IL-1β and IL-18. The role of P2X4 in various diseases could be beneficial or deleterious even though the pathophysiological mechanisms involved are still poorly defined. However, in diseases whose physiopathology involves activation of the NLRP3 inflammasome, P2X4 was found to exacerbate severity of disease. The recent production of monoclonal antibodies specific for the human and mouse P2X4, some of which are endowed with agonist or antagonist properties, raises the possibility that they could be used therapeutically. Analysis of single nucleotide polymorphisms of the human *P2RX4* gene has uncovered the association of *P2RX4* gene variants with susceptibility to several human diseases.

## Introduction

Extracellular adenosine triphosphate (ATP) is a neurotransmitter in the central and peripheral nervous system, and a mediator of inflammation in the immune system. It can also trigger a large variety of responses in different cell types that express various types of purinergic receptors (1, 2). These receptors belong to three different groups: P1, P2X and P2Y. Four P1 receptor subtypes (A_1_, A_2A_, A_2B_ and A_3_) are G-protein coupled receptors that bind adenosine, while eight human P2Y receptors (P2Y1,2,4,6,11,12,13,14) bind ATP or alternative purine nucleotides such as ADP (P2Y1,P2Y12,P2Y13), UTP (P2Y2,P2Y4), UDP (P2Y6,P2Y14) and UDP-glucose (P2Y14) (1–4). Seven P2X receptors (P2X1 to P2X7) are ATP-gated cation channels that can trigger numerous biological functions in normal and diseased tissues after ligation by extracellular ATP.

In this review, P2X4 is often compared to P2X7 because the two receptors are closely related (5) and they play an important role in immune cell activation.

## Structure of the P2X4 Receptor

**Figure 1:**
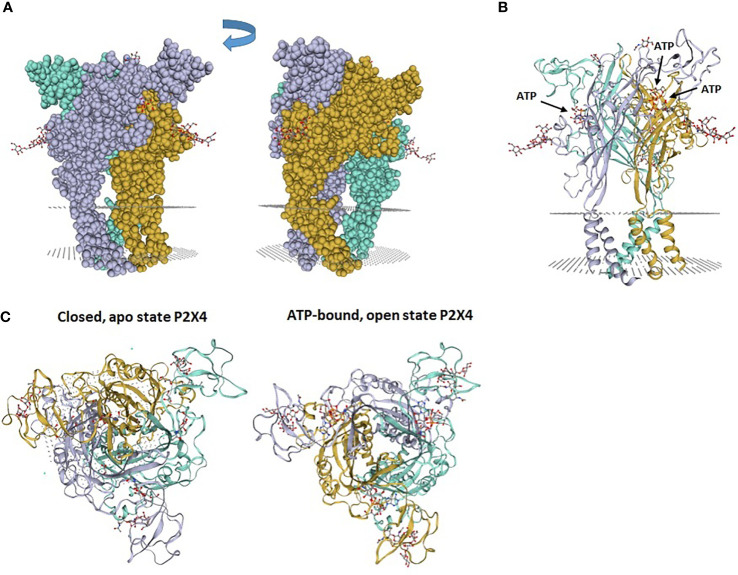
Zebrafish P2X4 receptor structure. **(A)** The P2X4 receptor is composed of 3 “dolphin”-like subunits, comprising a body, head, dorsal fin, right and left flippers and tail. Each “dolphin”-like subunit is shown in a different color (purple, yellow and blue). **(B)** Crystal structure of zebrafish P2X4 receptor showing 3 subunits (in purple, yellow and blue). Each subunit has transmembrane (TM)1 and TM2 α-helices and long ectodomains. The gray dotted areas in **(A, B)** indicate the presumptive plasma membrane location in which the receptor is inserted. **(C)** View from above of the crystal structure of zebrafish P2X4 receptor showing a closed, apo state P2X4 receptor and an ATP-bound, open state P2X4 receptor. Images (PDB ID 4DW0 and 4DW1) were obtained from the SWISS-MODEL Repository (10). 4DW0 indicates crystal structure of the ATP-gated P2X4 ion channel in the closed, apo state at 2.9 Angstroms. 4DW1 shows crystal structure of the ATP-gated P2X4 ion channel in the ATP-bound, open state at 2.8 Angstroms.

Cloning of the seven P2X receptors and the determination of crystal structures of several P2X have vastly improved our knowledge of P2X structure-functions relationships (6). The P2X receptor structure consists of three subunits that form a stretched trimer, supporting three ATP-binding sites (1). P2X receptor subunits associate to form homo- or heterotrimers, which differ by their electrophysiological and/or pharmacological properties (7). Each subunit has intracellular N- and C-termini linked by two transmembrane helices (TM1 and TM2) to a large extracellular ectodomain, which forms an ATP-binding pocket with another P2X ectodomain. The determination of crystal structures of an N- and C- termini truncated zebrafish P2X4 receptor without ATP (8) or bound to ATP (9) revealed for each subunit an original new fold called “dolphin”, comprising a body, head dorsal fin, right and left flippers and tail corresponding to the transmembrane helices (**Figure 1A**). The latter are structured as α-helices with the TM1 located outside the TM2, which delineates the ion-conducting pore (**Figure 1B**). The binding of extracellular ATP to P2X receptors induces the opening of the receptor allowing the entry of Ca^2+^ and Na^+^ and the efflux of K^+^ (**Figure 1C**). Schematically, the P2X receptors differ by their sensitivity for ATP and their kinetics of desensitization. Thus, P2X1 and P2X3 have the highest sensitivity for ATP (EC_50_ = 1 μM) and fully desensitize rapidly; while P2X2, P2X4 and P2X5 receptors have an intermediate sensitivity for ATP (EC_50_ ≅ 3-10 μM) and desensitization. The P2X7 receptor differs prominently from the other P2X receptors in that it is activated at higher concentrations of ATP (EC_50_ = 0.5-1 mM) and does not desensitize.

**Figure 2:**
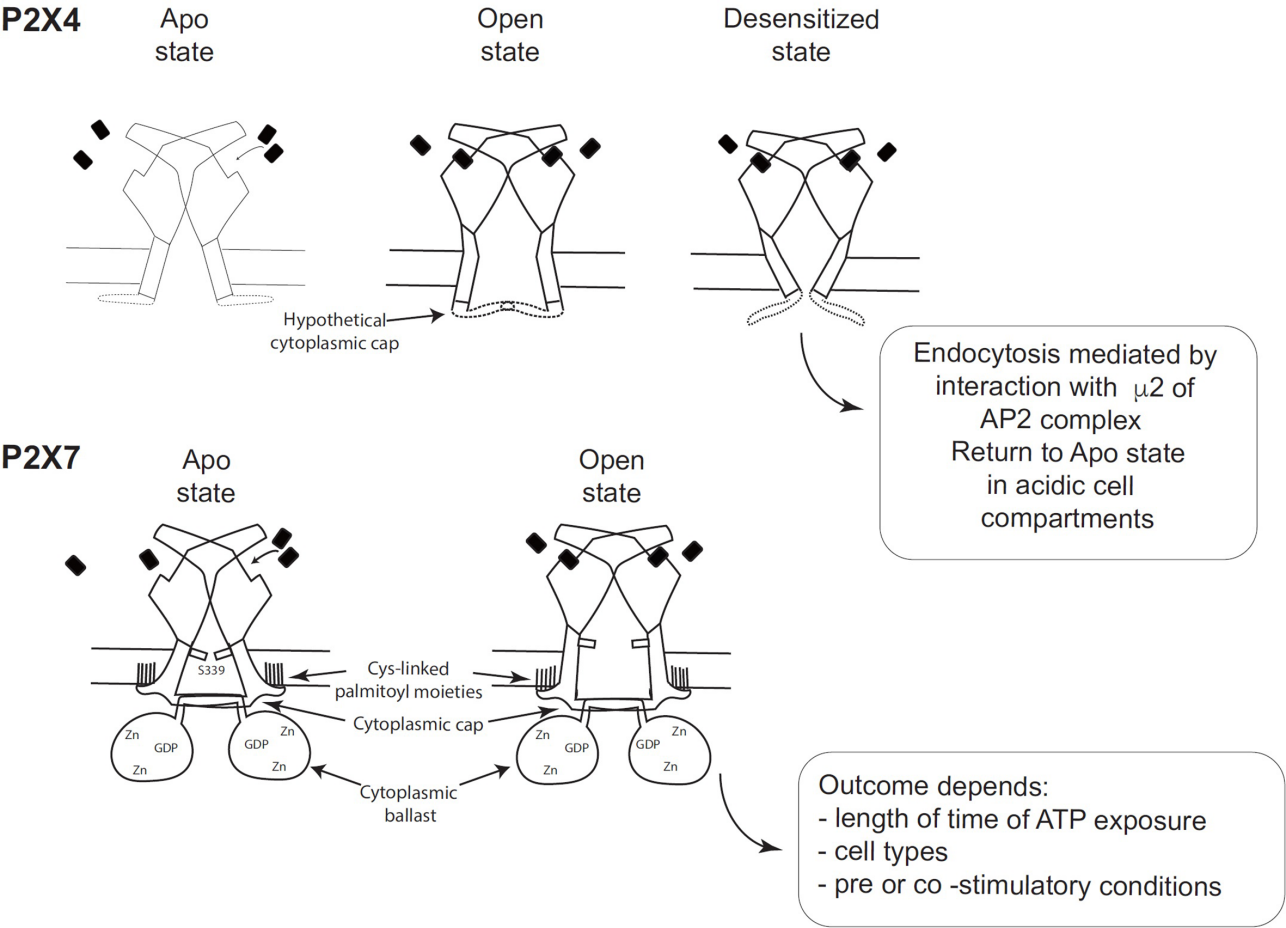
Schematic comparison of the desensitizing P2X4 receptor with the non-desensitizing P2X7 receptor (adapted from Figure 7 of McCarthy et al. (11). (Top panel) Hypothetical schematic figure of the P2X4 receptor in Apo state, Open state and Desensitized state. (Bottom panel) P2X7 receptor in Apo state and Open state. Black rectangles symbolize ATP molecules. As shown by McCarthy et al., the lack of desensitization of P2X7 is due to the stability of the cytoplasmic cap and to the palmitoylated residues of the C-cysteine anchors, which should stabilize the cytoplasmic cap and maintain TM2 in an open configuration (11).

The three ATP binding sites of a homotrimeric P2X4 were identified by analysis of the crystallographic structures (9). Each binding pocket is nested between the upper domain of one subunit and the lower domain of the adjacent subunit (**Figure 2**). The bound ATP molecule has a U-shaped structure and the negatively charged phosphate groups form salt bridge and hydrogen bonds with several basic and polar residues from the upper and lower domains of the two subunits. The adenine of ATP is deeply inserted into the ATP-binding site and forms three hydrogen bonds with the side chain of T189 and the carbonyl oxygen atoms of K70 and T189 (9). Additional hydrophobic interactions occur between adenine and leucine 191 in the lower body and isoleucine 232 of the lower fin. The ribose of ATP interacts with leucine 217 in the dorsal fin by hydrophobic interactions. For more details and an illustration of the ATP binding site, we refer the readers to the excellent article by Hattori et al. (9).

The fate of desensitized P2X4 was shown to depend on its recycling from the plasma membrane to intracytoplasmic acidic organelles (12). Convincing studies demonstrated that P2X4 is re-sensitized at acidic pH and that P2X4 re-sensitization is due to protonation/deprotonation of histidines in the extracellular domain. However, re-sensitization of P2X4 receptor at acidic pH is inhibited in the presence of ATP and is dependent on ATP concentrations (12). In lysosomes, the low pH (pH 5) should facilitate the release of bound ATP from P2X4 but the high concentrations of ATP in these organelles may block this process. Thus, additional mechanism(s) such as spatial or functional segregation might intervene to protect ATP-P2X4 complexes from lysosomal ATP (12).

## Structure/Function Relationships of Different P2X Receptors

Analysis of the structure of the human P2X3 receptor (13) and the rat P2X7 receptor (11), determined by X-ray and cryo-electron microscopy, respectively, unraveled several characteristic structural features of P2X3 and P2X7 without ATP (closed pore), with ATP-bound (open pore), and with ATP-bound (closed pore) but desensitized. These structures were very informative because they allowed the visualization of cytoplasmic residues which were truncated in the apo and ATP-bound structures of zebrafish P2X4 (9). Differences between the closed and open state of P2X3 showed that the β-0 and β-1 strands of the N-terminus of one protomer and the beta sheet of the C-terminus of the next protomer interact to create a “cytoplasmic cap” involved in the maintenance of the open state. This cytoplasmic cap was found only in the ATP-bound open state of P2X3, indicating that it is unfolded in the apo P2X3 and desensitized P2X3 states. The opening of the pore is mediated by a rotation of the TM2 domain in an “iris-like movement”, with a change in TM2 from an α-helix to a 3_10_ helix (13). Importantly, the transition from the open to the desensitized state results in the unfolding of the cytoplasmic cap, the recoiling of the TM2 domain upward, and the closing of the pore (13). This was described by Mansoor et al. (13) as the “helical recoil” model of receptor desensitization. The recent elucidation of the full length P2X7 receptor structure was a major breakthrough and revealed the overall organization of the cytoplasmic region and the functions of various domains (11). In particular, the lack of desensitization of P2X7 after ATP-binding is explained by the stability of the cytoplasmic cap formed by the β−0/β-1 sheet located at the N-terminus of each promoter in interaction with the β 15 sheet of the C-terminal end of the other protomer (11). Thus, in the ATP-bound P2X7 receptor, the three β-sheets of the cap overlay the open cytoplasmic pore delimited by the three TM2 regions. The stability of the cytoplasmic cap, compared with its counterpart in human P2X3, was found to be due to a region of P2X7 containing palmitoyl moieties: 4 cysteines (C362, C363, C374 and C377) and one serine (S360) (11). McCarthy et al. (11) showed that the palmitoylated residues are required for precluding P2X7 desensitization (**Figure 2**). They performed two different experiments, in which they deleted the 18-amino-acid long C-cysteine anchor from P2X7 or mutated all palmitoylable residues to alanine to preclude palmitoylation of the receptor. The modified P2X7 constructs were then expressed in *Xenopus* oocytes and compared to wild-type P2X7 receptor after stimulation with ATP. The two forms of modified P2X7 desensitized nearly completely while the wild-type receptor did not (11). Thus, the palmitoylated residues of the C-cysteine anchor play a major role in preventing desensitization of P2X7 (**Figure 2**). In the crystal structure, the palmitoylated moieties bound to 4 cysteines (C362, C363, C374 and C377) and one serine (S360) in the C-cysteine anchor and to one cysteine (C4) at the N terminus to anchor these segments into the intracytoplasmic face of the plasma membrane, which should stabilize the cytoplasmic cap and maintain TM2 in the open configuration (11). Thus, cytosolic cap stability plays a major role in lack of desensitization of P2X receptors (**Figure 2**). Since P2X4 has a pattern of desensitization intermediate between P2X3 (rapid) and P2X7 (no desensitization), one may hypothesize that the potential P2X4 cap stability is between these two patterns (**Figure 2**). However, structural information on the existence of a P2X4 cytoplasmic cap is missing, as X-ray structures of P2X4 were obtained with a truncated P2X4 receptor lacking the intracellular N- and C-terminal regions involved in cap formation.

Another major finding in the cytoplasmic part of the P2X7 structure corresponds to a novel original fold called the ballast domain (11), which comprises a dinuclear zinc ion complex and a high affinity binding site for GDP/GTP (**Figure 2**). Importantly, P2X7 construct lacking the ballast domain was shown to have gating properties identical to wild-type P2X7, demonstrating that the ballast domain is not involved in cation channel activity (11). However, the C-terminal region of P2X7 controls several biochemical pathways triggered after P2X7 stimulation [reviewed in Costa-Junior et al. (14)] and interacts with various proteins [reviewed in (15, 16)]. The ballast domain encoded by exon 13 was only found in the P2X7 receptor and in none of the other P2X receptors.

## Pores Associated With P2X Receptors

Upon prolonged ATP stimulation, several P2X receptors lead to opening of a large non-selective membrane pore that is permeable to molecules of molecular mass up to 900 Daltons. Two hypotheses have been proposed to explain pore formation: (1) P2X receptors possess an intrinsic capacity to dilate and form the pores; (2) pore formation involves additional molecules such as membrane hemichannels, i.e. pannexin-1 (17), connexin 43 (18) or anoctamin 6 (19) for the P2X7 receptor. However, in mice deficient for pannexin-1 or connexin 43, stimulation of P2X7 still triggers the formation of non-selective pores, showing that these hemichannels of macrophages are dispensable (20). The current controversies with regards to the permeability to large cations of P2X7, P2X2 and P2X3 following ATP stimulation have been recently reviewed elsewhere (21).

Three P2X receptors have the capacity to open non-selective pores after prolonged ATP stimulation, P2X2 (22–24), P2X4 (22–24), and P2X7 (25). Two teams (22–24) studied P2X4 receptors stimulated by ATP, in neurons or expressed heterogously in HEK cells or *Xenopus* oocytes. Khakh et al. (22, 23) found that after prolonged exposure to ATP, an initial current (I_1_) is followed by a slower current (I_2_), which developed quickly and reached a peak with an amplitude 150-300% of that of I_1_. In addition, they showed that I_2_ corresponded to an ion permeability increase of P2X4 receptors, allowing cations larger than Na^+^ to permeate into the cells. This increase in permeability was evidenced with studies using N-methyl-D-glucamine (NMDG^+^) or a propidium-dye, YO-PRO-1. Similarly, Virginio et al. (24) found that prolonged stimulation of P2X4 by ATP leads to an increase in permeability allowing cations as large as YO-PRO-1 (376 Daltons) to enter the cells. In contrast to P2X7, both groups found that P2X4-induced opening of the macropore was not associated with cell death. The results in favor of pore dilation of P2X receptors were obtained by patch-clamp electrophysiology (22–24) using NMDG^+^, a large synthetic organic cation, outside the cell, and Na^+^ inside. Brief stimulation of P2X4 receptors by ATP opens a pore that is more permeable to Na^+^ than NMDG^+^. During prolonged activation of P2X4 by ATP, reverse potentials (V_rev_) shift toward more positive values, indicating an increase in NMDG^+^ permeability. However, Li et al. (26) demonstrated that changes in V_rev_ measured in bi-ionic $NMDG_{out}^{+}/Na_{in}^{+}$ solutions were not due to a time-dependent increase in channel permeability but to a large modification in intracellular ion concentrations, i.e. a decrease in intracellular Na^+^ and accumulation of NMDG^+^ from 0 mM to 200 mM. While these studies challenge the pore dilation model, they show that the channels are permeable to large cations like NMDG^+^.

In primary murine microglia and murine microglial cells, prolonged exposure to 100 μM ATP stimulates the opening of a large P2X4 pore (27). Since P2X4 and P2X7 are expressed by murine microglial cells, the authors compared the P2X4-dependent and P2X7-dependent macropores using pharmacological inhibitors and ivermectin, an allosteric positive potentiator of P2X4. They found that formation of both macropores in microglia leads to YO-PRO-1 uptake, but only P2X7 activation triggered membrane blebbing and cytolytic death (27). LPS-stimulated macrophages from wild-type and P2X7-deficient mice were stimulated with 10 μM ATP or with 10 μM ATP plus 1-3 μM ivermectin (28), and the release of mature IL-1β was observed in the supernatants of macrophages from P2X7-deficient mice stimulated with ATP plus ivermectin only. These results demonstrate that P2X4 can also trigger this biochemical pathway in the absence of P2X7.

While numerous studies have been performed to identify the molecular components involved in the formation of the non-selective pore(s) following P2X7 stimulation, disagreements still remain (21, 25). In the case of P2X4, the preliminary evidence for the formation of a non-selective, non cytotoxic pore requires additional experimental proof.

## Expression and Trafficking of the P2X4 Receptor

Trafficking of the P2X4 receptor between the plasma membrane and endo-cellular compartments has been studied by several groups. Recently, the role of the P2X4 receptor in lysosomal membrane traffic and fusion has been thoroughly reviewed (29). Royle et al. (30) have identified a motif in the carboxy-terminus of P2X4 that controls the constitutive and agonist-regulated internalization of P2X4. When they mutated the following tyrosine-motif (Y378XXGL) of P2X4, they observed a decrease in receptor internalization associated with an increase in plasma membrane expression of P2X4 (30). They also compared the two tyrosine-motifs (Y372xxV and Y378xxGL) located within the C-terminus of P2X4. They found that the canonical motif (Y372xxV) is not used for endocytosis of the homomeric P2X4 receptor, while the mutation of the non-canonical one (Y378xxGL) efficiently diminished internalization (31). They also showed that a synthetic peptide corresponding to a sequence of P2X4 (VED**YEQGL**SG) co-crystallized with the μ2 subunit of the clathrin adaptor protein 2 (AP2), and binds to μ2 like the canonical YXXΦ motifs (31). Qureshi et al. (32) showed that P2X4 is preferentially localized in lysosomes in primary cells such as microglia, vascular endothelial cells and macrophages. In addition, they identified motifs in the N-and C-termini which are required to sort the P2X4 to lysosomes. The L22 and I23 mutations to alanine, at the N-terminus, increased the amount of P2X4 at the plasma membrane (32). Similarly, mutations of the tyrosine-motif (Y378XXGL) at the C-terminus of P2X4 decreased the levels of P2X4 in the lysosomal compartment. However, when the di-leucine and tyrosine motifs were mutated simultaneously, a major increase of P2X4 at the plasma membrane was observed, suggesting that the N- and C- termini participate in endocytosis and targeting of the P2X4 receptor (32). They also convincingly showed that P2X4 is protected from proteolysis by 6 N-linked complex oligosaccharides (32).

Interestingly, the lysosomal localization of P2X4 coexists with the presence of ATP in lysosomes of astrocytes (33), microglia (34) and hepatocytes (35).

Huang et al. (36) showed that the highest concentrations of ATP are found in lysosomes and mitochondria. In addition, using whole-lysosome patch clamp, these authors demonstrated that the lysosomal P2X4 is activated by luminal ATP when the pH was raised to 7.4 (36). These results suggest that, under physiological conditions, the high levels of ATP and P2X4 receptor in lysosomes do not lead to P2X4 activation because the lysosomal pH is between 3.5 and 5.0 and ATP is not in its active, tetra-anionic form at this low pH. These results are in agreement with the ATP-P2X4 receptor crystallographic structures showing that the α, β and γ negatively-charged phosphate groups of ATP interact with highly conserved basic and polar amino-acids of two subunits of P2X4 (9). The presence of high levels of ATP in lysosomes is due to the vesicular nucleotide transporter (VNUT)/SLC17A9, which is present in the lysosomal membrane and transports ATP across it (37). Silencing of VNUT/SLC17A9 in a mouse cell line induced a dramatic decrease of the ATP concentration in lysosomes, and the accumulation of lipofuscin in lysosomes, leading to cell death (37, 38). In addition, since P2X4 can be triggered by intra-lysosomal ATP as the pH increases (36, 37), Cao et al. (39) determined that P2X4 was the Ca^2+^-channel involved in the fusion of late endosomes and lysosomes. They showed that P2X4 stimulation released Ca^2+^, which activated calmodulin. As a consequence, calmodulin associated with P2X4 and promoted vesicle fusion (39).

An original strategy was recently developed by Xu et al. (40) to follow the cellular distribution of P2X4 among various compartments. They produced a pH-sensitive tagged P2X4 receptor by grafting the pHluorin protein within its extracellular domain (40). The P2X4-pHluorin construct was used to transfect HEK 293 cells, an immortalized microglial cell line, and primary hippocampal neurons and alveolar type-II cells. The authors incubated cells with 25 mM NH_4_Cl to increase the pH of acidic compartments, causing the fluorescent P2X4-pHluorin to become green (40). They could quantify the levels of P2X4 at the plasma membrane, in acidic compartments and within undefined additional compartments (40). While the relative amounts of P2X4-pHluorin at the plasma membrane and in acidic compartments depended on the cell type analyzed, acidic compartments always contained a larger fraction of P2X4 (40). This new methodology provides a powerful imaging tool to study the P2X4 cellular distribution and trafficking under various conditions.

## Monoclonal Anti-P2X4 Antibodies

Studies on P2X4 biology had been hampered by a lack of selective pharmacological inhibitors and monoclonal antibodies (mAb) against P2X4. However, both obstacles have now been overcome because various panels of mAbs (see **Table 1**) and good P2X4 inhibitors have become available, as reviewed elsewhere (46–48). We refer the readers to a recent review for more details on agonists, antagonists, positive allosteric modulators, negative modulators, as well as EC_50_ or IC_50_ in different species, and P2X cross-reactivity (48).

**Table 1:** Available antibodies against P2X4.

mAb or Nanobody	Mouse P2X4	Human P2X4	Rat P2X4	Cross-reacts with other P2X	Immunoprecipitation	Reacts with P2X4 in Western-blot	Activation or Inhibitionof P2X4 Activity	References
Mouse IgM mAb anti-P2X4			+++	No cross-reaction with all P2X (1,2,3,5,6,7)	Immunoprecipitates the rat P2X4	No	ND	Bo X. et al. (41)
Mouse mAb anti-P2X4		—	+++	ND	Immunoprecipitates the extracellular domain of rat P2X4	Reacts with rat P2X4	ND	Igawa et al. (42)
mAb 19 anti-P2X4 (IgG2b)	ND	+++			Immunoprecipitates human P2X4	No	No	Paalme et al. (43)
mAb 27 anti-P2X4 (IgG2b)	+++	+++	+++	Does not label P2X4 KO cells and does not react with human P2X7	Immunoprecipitates mouse and human P2X4	No	No	Paalme et al. (43)
mAb 29 anti-P2X4 (IgM)	+++	+++			Immunoprecipitates mouse and human P2X4	No	No	Paalme et al. (43)
IgG#151 LO	No	+++		No cross-reaction with all the other P2X			Inhibits human P2X4	Williams et al. (44)
IgG#191	+++						Inhibits P2X4. Reduces mechanical hyperalgesia	Williams et al. (44)
IgG#191-Bbbt0626	+++						Inhibits P2X4. Reduces mechanical hyperalgesia	Williams et al. (44)
Nodu 225 Mab Rat anti-P2X4	+++	—			Immunoprecipitates mouse P2X4 not human P2X4	No		Bergman et al. (45)
Nodu 246 mAb Rat anti- P2X4	+++	—			Immunoprecipitates mouse P2X4 not human P2X4	No	None	Bergman et al. (45)
Nodu 19 mAb Rat anti-P2X4	—	+++				No		Bergman et al. (45) Bergman et al. (45)
Nodu 344 mAb Rat anti-P2X4	—	+++				No		Bergman et al. (45)
Nb 262		+++				No		Bergman et al. (45)
Nb 284	+++	+++	+++			No		Bergman et al. (45)
Nb 318		+++	+++			No		Bergman et al. (45)
Nb 325	+++					No		Bergman et al. (45)
Nb 271	+++	+++	+++			No		Bergman et al. (45)
Nb 258	+++	+++				No		Bergman et al. (45)
Nb 301		+++		human & mouse P2X7		No		Bergman et al. (45)

In the following paragraph, we summarize the immunological, biochemical and functional characteristics of several monoclonal antibodies reacting with P2X4 from different species (**Table 1**). The first mAb described can recognize the extracellular domain (ECD) of rat P2X4 in its native conformation. This mAb immunoprecipitates a protein specifically recognized by polyclonal antibodies reacting with a C-terminal epitope of P2X4. This mAb reacts with P2X4 only and does not react with the other six P2X receptors (41). More recently, another mAb that recognizes the extracellular domain of rat P2X4 was produced after immunization of BALB/c mice with the refolded extracellular domain of rat P2X4. This mAb reacts in Western blots with the rat ECD of P2X4 and not with the human one (42). This mAb specifically recognizes and immunoprecipitates the rat P2X4 of transfected human astrocytoma. However, this mAb has not been tested in immunochemistry and flow cytometry assays (42). Paalme et al. (43) have produced several mouse mAb after immunization of C57BL/6 mice with the ECD of human P2X4. The three following mAb (mAb19, mAb27 and mAb 29 see **Table 1**) could immunoprecipitate the human and mouse P2X4 from lysates of HEK cells stably transfected with different constructs of human P2X4 or mouse P2X4 (43). Flow cytometry analysis of transfected cells, and immunoprecipitation and immunohistochemistry experiments demonstrated that these mAbs specifically recognize P2X4 in its native conformation. Flow cytometry analyses showed that mAb27 reacts with macrophages of wild-type animals and not with those from P2X4-deficient mice (43). In addition, mAb27 does not label HEK cells expressing the human P2X7 receptor, demonstrating its specificity for P2X4. Importantly, these mAb were used to characterize the expression of P2X4 receptor on mouse and human peripheral blood lymphocytes (PBL). The highest P2X4 surface expression level was found on eosinophils from PBL and could be a useful marker for this cell type in conjunction with Siglec-8. Using mAb27, the expression levels of P2X4 on PBL from human and mice were significantly higher on male leucocytes than on female ones. This sex difference in the two different species needs further investigation.

Williams et al. (44) have produced a large panel of mAb to human and murine P2X4 that have significant inhibitory or stimulatory properties. Among the inhibitory mAbs, one can block hP2X4 currents efficiently. This antibody, IgG #151, was subjected to mutagenesis of its VH and VL CDR3 loops, followed by several rounds of selection to obtain an optimized mAb with increased binding affinity. The optimized mAb, IgG #151 LO, blocked hP2X4 more efficiently than the original IgG #151. IgG #151 LO had an IC_50_ of 0.7 nM, while IgG #151 had an IC_50_ of 1.2 μM (44). Since none of the 6 anti-human P2X4 inhibitory mAb reacted with murine P2X4, the authors immunized rats with purified full length murine P2X4 receptor and HEK cells expressing mP2X4. After immunization, rat hybridomas were produced and 4000 clones were screened for specific binding to mP2X4. Among the 251 mAb that bound P2X4, 41 had inhibitory properties and 18 potentiated mP2X4 currents. The most powerful inhibitor, IgG #191, was selected and used to determine whether this mAb could decrease the mechanical hyperalgesia in a murine model of neuropathic pain (44). IgG #191 was injected subcutaneously or intrathecally into mice that underwent partial sciatic nerve ligation. Intrathecal injection of IgG #191 reduced significantly neuropathic pain, when compared with an isotype control IgG. Subcutaneous injections had no effect on hyperalgesia, suggesting that peripheral P2X4 receptors might not be implicated in this mouse model of pain.

Interestingly, the same authors had previously produced an antibody (BBBt0626) which can be transferred across the blood brain barrier (BBB) into the central nervous system (CNS). Thus, they created constructs of IgG #191 in which one scFV of Bbbt026 was linked to each C terminus of the CH3 domain of IgG#191. The IgG #191Bbbt026 fusion mAb was injected subcutaneously to different groups of mice, which suffered from neuropathic pain. A very significant dose-dependent reduction of hyperalgesia was observed in mice treated with IgG#191Bbbt026 and not in a control group injected with isotype control antibody fused to Bbbt026. This experiment shows that IgG#191Bbbt026 could cross the BBB and keep its anti-P2X4 inhibitory properties, and suggests a therapeutic potential as they can reverse neuropathic pain *in vivo* (44).

Using a DNA vaccine approach, Bergmann et al. (45) immunized rat and llamas to produce rat monoclonal antibodies and llama nanobodies reacting with human, murine and rat P2X4 receptor. They used an expression vector encoding the P2X4^Y378F^ mutant, which, unlike the wild-type receptor, has lost its ability to be internalized and thus is expressed at the cell surface on transfected cells. They were able to produce several rat anti-P2X4 mAbs, Nodu 225 and Nodu 246, which only react with murine P2X4 and two other mAbs (Nodu 19 and Nodu 344), which only bind to human P2X4. They fully characterized one of them (**Table 1**). Nodu 246 specifically reacted with mouse P2X4 in immunohistochemistry, flow cytometry and immunoprecipitation assays; it labeled various P2X4^+/+^ cells and immunoprecipitated P2X4 from P2X4^+/+^ bone marrow derived macrophages (BMDM), while Nodu 246 did not react with P2X4^-/-^ cells and lysates used as controls. Interestingly, Nodu 246 labeled weakly non-permeabilized BMDM but gave a very strong fluorescent signal if BMDM were permeabilized before addition of the fluorochrome-labeled mAb. This observation is in agreement with the intracytoplasmic lysosomal localization of P2X4. Seven anti-P2X4 nanobodies were obtained and the seven VHH were cloned in an expression vector to link directly the VHH to the hinge, CH2 and CH3 domains of rabbit IgG producing a divalent-nanobody rabbit IgG with increased avidity. Using HEK cells expressing human, rat or mouse P2X4 and human or murine P2X7 (as control of specificity), they showed that Nb262 and Nb301 react with human P2X4 only (**Table 1**). Two nanobodies (Nb271 and 284) bind to human, rat and mouse P2X4, while one (Nb 258) recognizes human and mouse P2X4 (**Table 1**).

Interestingly, a powerful inhibitor of P2X4 (IgG #191) has been used to treat some P2X4-dependent diseases. A similar strategy has been used by Danquah et al. (49) who have produced a llama nanobody with potent antagonist effect against P2X7. This nanobody inhibited P2X7 functions of macrophages and T lymphocytes and, when injected *in vivo*, decreased inflammation in two mouse disease models: experimental glomerulonephritis and allergic contact dermatitis (49).

## Physical Interactions Between P2X4 and P2X7

The study by Torres et al. (50) was the first to determine whether the seven members of the P2X family can associate as homomers or heteromers. For this purpose, they produced rat P2X subunits tagged with different epitopes and transfected them in HEK cells alone or associated as pairs to examine whether they could form homomers or heteromers, respectively. They found that all subunits except P2X6 can co-assemble to produce homomers. P2X7 also forms homomers but is unable to associate with any other P2X receptor. In contrast, P2X4 receptors form heteromers with P2X5 or P2X6. These conclusions were revisited by Guo et al. (51) who provided biochemical and electrophysiological data indicating that rat P2X4 and P2X7 can form heteromeric receptors. They co-expressed P2X4-tagged and P2X7 in HEK cells and immunoprecipitated P2X4 with an anti-tag antibody. They found that P2X7 was co-immunoprecipitated with P2X4. To eliminate an artifact due to overexpression of P2X4 and P2X7 in HEK, they also used BMDMs. Immunoprecipitation of P2X4 endogenous receptors with anti-P2X4 allowed them to detect P2X7 at the expected molecular weight in Western-blots with anti-P2X7 antibodies. They also provided additional evidence showing that when P2X4 and P2X7 were co-expressed, ivermectin, an allosteric modulator of P2X4, could confer ivermectin-responsiveness to BzATP, a ligand of P2X7. Furthermore, co-expression of P2X7 with P2X4 in HEK cells modified the responses observed with BzATP (P2X7 agonist) and MgATP (P2X4 agonist). When P2X4 or P2X7 were expressed alone in HEK cells, stimulation with BzATP or MgATP induced large currents in HEK-P2X7 or HEK-P2X4 cells, respectively. In contrast, in HEK cells expressing both receptors, the currents triggered by both agonists were diminished, strongly suggesting that P2X4 and P2X7 interacted functionally. In contrast, two separate studies, using rat or mouse P2X4 and P2X7, claimed that P2X4 and P2X7 do not co-assemble to produce P2X4/P2X7 heterotrimers (52, 53). P2X4 or P2X7 complexes from lysates of different tissues were separated on blue native (BN)-PAGE and detected by immunoblotting using anti-P2X4 and anti-P2X7 specific antibodies. The results showed that P2X4 and P2X7 are trimeric complexes but there was no evidence of P2X4/P2X7 heterotrimers. In addition, overexpression of P2X4 and P2X7 in *Xenopus* oocytes showed that P2X4/P2X7 heterotrimers were not found (52).

In agreement with these findings, using BN-PAGE and two different chemical cross-linkers, Boumechache et al. (53) studied the subunit content of P2X4 and P2X7 complexes expressed by primary cultures of rat microglia and macrophages from wild type and P2X7-deficient mice. They observed in cell lysates treated with disuccinimidyl suberate and plasma membrane cross-linked with a membrane-impermeant cross-linker that P2X4 or P2X7 complexes were homotrimers. While these two studies are convincing, one cannot completely rule out the possibility that a very small percentage of P2X4/P2X7 heterotrimers co-assemble but are not detected. As mouse parotid acinar cells express P2X4 and P2X7 receptors, a laboratory (54) compared the electrophysiological properties of ATP-stimulated currents in acinar cells with HEK293 cells transfected with mouse P2X4 or P2X7 cDNA or with different mixtures of P2X4/P2X7 cDNA ratios. They found that electrophysiological and pharmacological properties of P2X4- and P2X7-homomers expressed in HEK293 cells were different from those observed in HEK293 cells and in mouse acinar cells co-expressing P2X4/P2X7. These findings led the authors to hypothesize that the interaction of murine P2X4 and murine P2X7 is due to the assembly of heterotrimeric P2X4/P2X7 complexes with novel electrophysiological properties, to an interaction between homomeric P2X4 and P2X7 receptors, or a combination of both mechanisms.

More recently, the interaction of P2X4 and P2X7 subunits was reassessed using two different strategies: measurements of Förster resonance energy transfer (FRET), and analyses of ATP-activated ion currents in *Xenopus* oocytes transfected with human P2X4 or P2X7 or with both human P2X4 and P2X7. Thus, P2X4 and P2X7 subunits labeled with EGFP and TagRFP fluorescent proteins, respectively, were co-expressed in the oocytes. This allowed Schneider et al. (55) to determine whether a close physical interaction between P2X4 and P2X7 occurred. Significant FRET signals were measured with P2X4 and P2X7 labeled with EGFP or TagRFP in their extracellular domains or in their C-terminal regions. On the basis of these results, the authors concluded that P2X4 and P2X7 subunits were assembled as heterotrimers. Using the two-electrode voltage clamp technique, they then examined whether the human P2X4 and P2X7 subunits, co-transfected in *Xenopus* oocytes, produced a new electrophysiological phenotype due to a functional interaction. Surprisingly, they found that at low ATP concentrations only human P2X4 receptors were activated, while at high ATP concentrations, human P2X7, with low ATP affinity, became stimulated. In addition, the effects of P2X4- and P2X7-specific pharmacological drugs on oocytes co-expressing human P2X4 and human P2X7 strongly suggested that no functional interaction occurred and that co-expression of P2X4 and P2X7 does not generate a new electrophysiological phenotype different from that of P2X4 or P2X7 alone. More recently, Trang et al. (56) used murine microglial BV-2 cells and the whole cell voltage clamp technique to determine whether P2X4 and P2X7 receptors are activated independently or interact molecularly and/or functionally. Using the selective pharmacological P2X4 (PSB-15417) and P2X7 (A438079) inhibitors, they found strong inhibition of the current, demonstrating that no P2X receptors other than P2X4 and P2X7 are responsible for the ATP-dependent currents in BV-2 cells. They further analyzed P2X4 and P2X7 mediated current components with or without P2X4 and P2X7 receptor pharmacological inhibitors and compared the current kinetics. Their results showed that P2X4 and P2X7 are activated independently, strongly suggesting that P2X4/P2X7 heteromers are not formed. They also treated BV-2 cells with the pro-inflammatory molecules LPS and interferon-γ (IFN-γ) to induce an M1 phenotype *in vitro*. Only the simultaneous treatment with LPS and IFN-γ induced a significant increase in P2X4 current components, while P2X7 receptor dependent ones remained unchanged. These results also argue against a model in which physical or functional heteromerization of P2X4 and P2X7 receptor protomers takes place.

Importantly, the studies of Antonio et al. (57) suggested that interpretation of biochemical results indicating that heterotrimers of P2X4/P2X7 exist requires additional tests such as chemical cross-linking experiments confirming heteromer formation and atomic force microscopy (AFM) imaging. Indeed, in their studies, a close interaction between rat P2X4 and P2X7 was suggested in cells using the *in situ* proximity ligation assay and by co-immunoprecipitation from detergent lysates or co-purification by affinity (57). However, when detergent lysates of membranes from cells co-transfected with rat P2X4-HA and rat His_10_-P2X7 were treated with the chemical cross-linker disuccinimidyl suberate, immunoprecipitation with anti-HA antibody did not reveal the presence of heterotrimers. Moreover, AFM was used to generate images of single receptors isolated from cells expressing P2X4 or P2X7 and P2X4/P2X7. The results of AFM experiments indicated that interaction between P2X4 and P2X7 was due to association of homotrimers and not to the production of heterotrimers.

Presently, despite great efforts to determine whether P2X4 and P2X7 interact as heterotrimers or homotrimers, there is no definitive proof in favor of one model or the other. However, several publications show that functional interactions between P2X4 and P2X7 do occur (see below).

## Functional Interactions Between P2X4 and P2X7

Kawano et al. (58) have studied the role of P2X4 in P2X7-dependent cell death of RAW264.7 mouse macrophages. They found that stimulation of P2X7 induces a calcium influx, non-selective pore formation, phosphorylation of MAPKinase ERK1/2 and p38, and cell death. Using sh-RNA to silence P2X4, ATP stimulation of RAW264.7 cells leads to a strong decrease in the early burst of Ca^2+^ influx and a significant inhibition of cell death, while non-selective pore formation and MAPkinase activation were not affected. Since stimulation of P2X4 alone does not trigger cell death, these results strongly suggest that co-expression of P2X4 with P2X7 promotes P2X7-dependent macrophage death. Further studies on RAW264.7 cells confirmed that P2X4 potentiates P2X7-dependent release of IL-1β and HMGB1 through an early increase in Ca^2+^ influx boosting the Ca^2+^ influx following P2X7 stimulation (58). These studies in a murine macrophage cell line were extended to BMDC (59). In LPS-stimulated BMDC, the stimulation of P2X7 by ATP triggers NLRP3-dependent activation of caspase 1, which is involved in the proteolytic cleavage of pro-inflammatory cytokines IL-1β and IL-18. BMDC express P2X4 and P2X7 which, after ATP stimulation, triggers Ca^2+^ influx. The sh-RNA silencing of P2X4 induced a significant decrease in the Ca^2+^ influx and a down-modulation of IL-1β and IL-18 release (59). Overall, these studies suggest that P2X4 can positively control P2X7-dependent Ca^2+^ influxes and potentiates inflammation *via* P2X7-dependent maturation of pro-inflammatory cytokines.

Interestingly, the role of P2X4 in an ovalbumin (OVA)-driven model of allergen-induced airway inflammation (AAI) was studied by Zech et al. (60), who showed that P2X4 expression in the lung increased in mice suffering from AAI as compared with control littermates. This increase in P2X4 was also found in cells in the broncho-alveolar lavage fluid (BALF) of asthmatic individuals compared with healthy individuals. The involvement of P2X4 in AAI was clearly established because treatment of sensitized mice with the selective P2X4 pharmacological inhibitor 5-BDBD (benzodiazepine derivative, 5-(3-bromophenyl)-1,3-dihydro-2H-benzofuro[3,2-e]-1,4-diazepin-2-one) decreased inflammation, and P2X4-deficient mice developed a less severe AAI compared with wild-type controls. Thus, in 5-BDBD treated mice and in P2X4-deficient animals, a significant reduction of the number of BALF cells was found as well as a decrease in production of IL-4, IL-5 and IL-13 by mediastinal lymph node cells after allergen boost (60). ATP stimulation of allergen sensitized BMDCs of WT mice triggered the release of IL-1β as expected, but in BMDCs from P2X4-deficient animals, a significant decrease of IL-1β production was observed. Interestingly, in allergen-sensitized wild-type BMDCs, there was a significant increase in P2X7 mRNA expression, which is not found in BMDCs from P2X4-deficient mice. Thus, these results suggest that P2X4, in addition to its effect on early burst of Ca^2+^ influx, triggers upregulation of P2X7, which contributes to increased production of IL-1β (60).

## Role of P2X4 in Mast Cell Degranulation

Mast cells are bone-marrow derived cells involved in allergic inflammatory reactions and type I hypersensitivity responses. These cells contain large granules which, after exocytosis, release numerous chemical mediators involved in allergic reactions such as serotonin, histamine, heparin and various proteases. Yoshida et al. (61) have studied the role of P2X4 in mouse bone-marrow derived mast-cell (BBMC) degranulation. They showed that BBMC express several P2 ionotropic receptors such as P2X1, P2X4 and P2X7. Among these receptors, only P2X7 can trigger degranulation upon ATP stimulation. This effect is P2X7-specific because it could be blocked by pre-incubation of mast cells with the P2X7 pharmacological inhibitor, AZ10606120. Mast-cells expressing the high affinity receptor for IgE can bind antigen-specific IgE which, after cross-linking by multivalent antigen, induces rapid degranulation. Interestingly, P2X4 stimulation by low doses of ATP does not trigger degranulation directly but potentiates the BBMC degranulation induced by cross-linking of the high-affinity IgE receptor (FcϵRI). The ATP-potentiation of degranulation is blocked by the P2X4-specific pharmacological inhibitor, 5-BDBD, or by P2X4-specific siRNA knock-down in BBMC. In recent studies, Yoshida et al. (62) evaluated the role of P2X4 in mast-cell degranulation triggered by cross-linking of IgE-FcϵRI complexes by antigen using BBMC of wild-type and P2X4-deficient mice. They found that ATP stimulation of wild-type BBMC potentiates antigen-triggered degranulation, while this effect was strongly reduced in BBMC from P2X4-deficient mice. Importantly, the potentiating effect of ATP is recovered when P2X4-deficient BBMC are transfected with a P2X4-encoding plasmid. Cross-linking IgE-FcϵRI complexes by antigen leads to the recruitment and activation of the tyrosine-kinase Syk which triggers the phosphorylation of phospholipase Cγ (PLCγ) and Akt. Yoshida et al. (62) found that ATP stimulation enhanced antigen-induced phosphorylation of Syk and PLCγ in BBMC from wild-type mice, while this phenomenon was diminished in P2X4-deficient BBMC. Since stimulation of BBMC by ATP alone did not activate Syk and PLCγ, the potentiating effect of ATP must be mediated by P2X4. Intriguingly, the potentiating effect of P2X4 on phosphorylation of Syk and PLCγ was found to be independent of Ca^2+^ influx (62). This raises the following question: how can P2X4 activation potentiate FcϵRI phosphorylation cascades independently of ion channel activity?

## Role of P2X4 Receptor in Inflammasome Activation and Inflammation

Inflammasomes are multi-protein cytoplasmic platforms that assemble in the host-cell cytosol after detecting stress, infection and sterile insults. Activation of inflammasomes can lead to maturation and secretion of the pro-inflammatory cytokines IL-1β and IL-18, and activation of an inflammatory form of cell death called pyroptosis (63). There are several inflammasomes, which are named after the pathogen recognition receptor (PRR) involved in detection of the stress/infection, such as NLRP3, NLRP1b, AIM2 and NLRC4. The NLRP3 inflammasome is the most studied inflammasome and typically requires two signals for its activation. Signal 1 requires stimulation by a pathogen-associated molecular pattern (PAMP), such as LPS, which leads to transcription of NF-κB and upregulation of genes encoding pro-inflammatory cytokines and chemokines, as well as proteins involved in inflammasome assembly. Signal 2 requires stimulation by a danger-associated molecular pattern (DAMP), such as HMGB1, IL-1α or extracellular ATP, which results in inflammasome activation (64). We and others have extensively studied the effects of extracellular ATP on the NLRP3 inflammasome activation through P2X7 receptor signaling (65–68). A number of downstream pathways from P2X7 ligation have been described, including efflux of K^+^ and production of reactive oxygen species (ROS) following ligation of P2X7 by ATP (69, 70). In this review, we will focus our discussion on mechanisms whereby P2X4 may modulate inflammasome activation in different tissues.

Our group and others previously reported that P2X4 interacts with P2X7 and shares some functional features with P2X7 (58, 59). We demonstrated that the P2X4 receptor is functionally associated with the P2X7 receptor and pannexin-1 in gingival epithelial cells (GEC) and modulates ATP-induced ROS production (70). After infection of GEC with *Porphyromonas gingivalis*, ATP-induced IL-1β secretion was inhibited by P2X4 receptor antagonists and P2X4 depletion. This suggests that P2X4, by physically interacting with P2X7 and pannexin-1, modulates NLRP3 inflammasome activation.

Since P2X4 is expressed in several tissues, P2X4 could conceivably modulate inflammasome activation in different tissues and clinical conditions. In rat urothelial cell line (MYP3 cells), hydrostatic pressure induced ATP release and intracellular caspase-1 activation (71). Treatment with P2X4 antagonists had no effect on ATP release but it inhibited pressure-induced caspase-1 activation. Moreover, the P2X4 receptor antagonist also inhibited ATP-induced caspase-1 activation in MYP3 cells, suggesting that the inflammasome may be activated through P2X4 receptor in urothelial cells (71). A recent study showed that the P2X4 receptor exacerbated ischemic acute kidney injury through activation of the NLRP3 inflammasome (72). In this study, P2X4-deficient mice showed significantly attenuated NLRP3 inflammasome signaling after renal ischemia and reperfusion, compared with wild-type controls. Using human proximal tubule cells and proximal tubule cells derived from wild-type or P2X4-deficient mice, the authors also demonstrated that P2X4 agonists induced expression of IL-1β and NLRP3 mRNA, while a P2X4 antagonist inhibited ATPγS-induced NLRP3 and IL-1β mRNA expression (72). Another study demonstrated that heme, the hemoglobin component that is released when hemolysis or extensive cell damage occurs, activates the NLRP3 inflammasome in macrophages through P2X7 and P2X4 (73). Using gene-silencing techniques, the authors showed that heme activated caspase-1 and IL-1β through P2X7 and P2X4 (73). Another group demonstrated that P2X4 and NLRP3 were highly expressed and these two proteins co-localized in renal tubule cells of type 2 diabetic patients with nephropathy (74). The authors also showed that the P2X4 signaling pathway mediated NLRP3 inflammasome expression and caspase-1/IL-1β/IL-18 production in HK-2 cells (an immortalized proximal tubule epithelial cell line from human kidney) (74). These studies suggest that, not only the P2X7 receptor, but also the P2X4 signaling pathway can activate the NLRP3 inflammasome in the kidneys and that P2X4 may be an important therapeutic target in renal disease.

In a mouse model of spinal cord injury, P2X4-deficient mice presented impaired inflammasome signaling in the spinal cord, which resulted in decreased levels of caspase-1 and IL-1β, but not IL-18 (75). The P2X4-deficient animals also showed decreased inflammatory cell infiltration and an improvement in functional outcomes after spinal cord injury. A separate study showed that the envelope glycoprotein 120 (gp120) of human immunodeficiency virus type 1 (HIV-1) induced pyroptotic cell death, IL-1β and IL-18 secretion, as well as an increase in expression of NLRP1 and P2X4 in satellite glial cells of dorsal root ganglia (76). Depletion of P2X4 decreased all these inflammasome measurements, suggesting that gp-120 can activate the inflammasome in the cells of the central nervous system through P2X4 (76). Comparing microglia and BMDM, it was found that ATP-induced IL-1β secretion in BMDM was fully dependent on P2X7 signaling, while in microglia, multiple purinergic receptors were involved, including P2X4 and P2X7 (77). These studies suggest that P2X4 may be a possible therapeutic target during neurodegenerative disorders.

In a mouse model of arthritis, P2X4 inhibition led to a decrease in caspase-1, ASC, and NLRP1 and consequently a decrease in IL-1β production (78). The authors suggested that the P2X4 inhibitor attenuated the severity of arthritis in mice due to inhibition of the NLRP1 inflammasome.

Taken together, these results suggest that P2X4 may play an important role in inflammasome activation in several organs and systems of the body including the central nervous system, oral cavity, kidneys and blood cells, even if it does so to a lesser extent than the P2X7 receptor. The P2X4 receptor may therefore serve as a therapeutic target for several clinical conditions involving inflammasome activation.

P2X4 also has other functions associated with inflammation. P2X4 triggers the secretion of inflammatory mediators including two pain inducers, prostaglandin E2 (PGE2) and brain-derived neurotropic factor (BDNF) (79, 80). Both of these pain signaling pathways are therefore linked to increased cell surface expression of P2X4. In particular, P2X4 mediates PGE2 release by tissue-resident macrophages. During PGE2 biosynthesis, arachidonic acid (AA) is released from membranes by phospholipase A2 (PLA2), a reaction controlled by calcium, hence potentially by ATP and P2X4. Interestingly, P2X4-deficient mice show a complete absence of inflammatory PGE2 in tissue exudates, indicating that P2X4 may be the main trigger of inflammation-induced PGE2 synthesis *in vivo* (81). In fact, both neuropathic and inflammatory pain are correlated with P2X4 expression at the cell surface of microglia/macrophages. Interestingly, an increased surface density of P2X4 receptors on neurons reduces anxiety and affects spatial memory, as shown using a conditional transgenic knock-in P2X4 mouse line (82).

P2X4 is also involved in neutrophil chemoattraction. In macrophages, ATP is one of the danger molecules that induce intercellular P2 receptor signaling. Thus, P2X4 modulates ATP-induced macrophage CXCL5 expression and secretion, which is important for neutrophil recruitment to the inflammation site (83). Moreover, while platelets are the main source of CXCL5 under homeostatic conditions, CXCL5 is also produced by lung epithelial cells in order to attract neutrophils. In humans, CXCL5 is a proinflammatory chemokine that regulates CXCR2-dependent neutrophil trafficking and directed migration.

## The P2X4 Receptor and Sepsis

Sepsis is a life-threatening organ dysfunction resulting from dysregulated host responses to infection (84). This syndrome is one of the leading causes of deaths globally, and represents a major public health issue with considerable economic consequences (85). In 2017, the World Health Organization defined prevention and treatment of sepsis a global health priority, given that the management of this syndrome is complex, including restoration of tissue perfusion *via* fluid administration and antimicrobial therapy (85). Increased plasma ATP levels found in mouse model for sepsis (86) and patients with sepsis suggests that purinergic receptors may be ligated and play a role during this condition (87). Here, we discuss what is known about the involvement of P2X4 in sepsis.

The first study to describe the role of P2X4 during sepsis used the α-hemolysin-producing *Escherichia coli* mouse infection model of sepsis. The authors demonstrated that P2X7-deficient or P2X4-deficient mice had lower survival rates, higher cytokine levels and activated intravascular coagulation, compared with wild-type controls (88). To explain the high levels of cytokines, including IL-1β, in the receptor-deficient mice, the authors showed that cytokine production was dependent on caspase-8 and RIPK3 as an alternative to the P2X7-dependent caspase-1 pathway. This study suggested that activation of P2X4 and P2X7 plays a protective effect against sepsis during infection with uropathogenic *E. coli* (88).

A second study addressed the role of P2X4 in the sepsis mouse model of cecal ligation and puncture (CLP) (89). In this study, P2X4-deficient mice showed a lower survival rate, increased bacterial burden and higher level of inflammatory cytokines and chemokines in the blood and peritoneum compared with wild-type controls (89). In addition, they produced mice in which the P2X4 gene was specifically deleted in myeloid cells, and found that in these mice sepsis was more severe with increased bacterial burden and elevated cytokine levels when compared with control mice. This idea is supported by *in vitro* data showing that ATP-induced *E. coli* killing in infected macrophages depends on P2X4 through mitochondrial ROS production (89). These data suggest that P2X4 in macrophages is protective and suppresses bacterial dissemination and inflammation *in vivo*.

## Role of P2X4 in T Cell Receptor Signaling and T Cell Migration

Several studies have shown that T cell receptor (TCR) stimulation triggers ATP release in the extracellular milieu through plasma membrane hemi-channels such as pannexin 1 on T lymphocytes (90, 91). Several P2X receptors are expressed on T lymphocytes: P2X7 (90, 91), P2X1, and P2X4 (92). Woehrle et al. (92) studied the role of P2X receptors in the control of T cell activation following TCR stimulation induced by anti-CD3 and anti-CD28 antibodies coupled to microbeads. They observed that P2X1 and P2X4 translocate rapidly to the immune synapse, formed by T cells and microbeads, and persist at the synapse for 1 hour after cell stimulation. In contrast, P2X7 receptors do not translocate to the synapse and remain evenly distributed at the plasma membrane. When cells from the human T cell leukemia line Jurkat are stimulated by anti-CD3 and anti-CD28 antibodies, siRNA silencing of P2X1 or P2X4 or P2X7 receptors inhibits Ca^2+^ signaling. Using pharmacological inhibitors of P2X1 and P2X4, they also found that Ca^2+^ signaling was inhibited in human primary CD4^+^ T cells triggered by anti-CD3/CD28 antibodies. Interestingly, they showed that P2X1 and P2X4 are involved in NFAT activation and interleukin-2 production after TCR stimulation. However, pharmacological inhibition of P2X4 always induces a higher inhibition of NFAT and IL-2 production than P2X1 inhibition by NF023 (a selective inhibitor of P2X1). It is worth noting that inhibition of P2X7 receptors was shown to decrease Ca^2+^ signaling and IL-2 transcription by two different laboratories (90, 91). Thus, P2X1, P2X4, and P2X7 receptors participate in TCR-mediated T lymphocyte activation. Interestingly, Woehrle et al. (92) observed that pannexin-1 translocates to the immunological synapse very quickly after TCR/CD28 stimulation, while it is evenly distributed at the plasma membrane on resting T lymphocytes. Furthermore, pharmacological inhibition of pannexin-1 decreased Ca^2+^ signaling and IL-2 transcription in response to TCR stimulation. Thus, Woehrle et al. (92) argue that, since P2X1 and P2X4 are found at the immunological synapse with pannexin-1, these receptors are preferentially involved in T cell activation. This interpretation is strengthened by their observation that STIM1 and Orai1 are present at the immunological synapse with P2X1, P2X4, and pannexin-1 (92). STIM1 and Orai1 are molecular constituents of CRAC channels, which play a major role in T cell activation (93, 94). Overall these data, obtained mostly with human T lymphocytes or Jurkat cells stimulated by anti-CD3/CD28 antibodies coupled to microbeads, showed that TCR stimulation is amplified by purinergic signaling at the immunological synapse.

Ledderose et al. (95) identified the endocellular origin of ATP released after TCR stimulation on T lymphocytes by anti-CD3/CD28 antibody-coated beads. They showed that increased production of ATP by mitochondria occurred rapidly after TCR simulation, and that activated mitochondria translocated to the immune synapse (95). Thus, the amplification of TCR signals at the immune synapse results from the accumulation of ATP-producing mitochondria, the presence of pannexin-1 involved in ATP release, and of P2X1 and P2X4 receptors which increase Ca^2+^ influx in response to ATP. More recently, Ledderose et al. (96) studied the role of the chemokine CXCL12 and its impact on purinergic signaling and TCR stimulation. CXCL12 binds to the chemokine receptor CXCR4, which is expressed on T lymphocytes. They found that CXCL12 stimulation of naive CD4^+^ T cells triggers cell migration and rapid release of ATP into the extracellular space. siRNA-silencing of Pannexin-1 in Jurkat T cells abolished cell migration and ATP release (96). In addition, blocking of mitochondria with carbonyl cyanide m-chlorophenyl hydrazine, an inhibitor of oxidative phosphorylation, inhibited ATP release and migration of T cells after CXCL12 stimulation. Furthermore, they showed, using sophisticated live-cell imaging, that mitochondrial Ca^2+^ uptake and ATP release were localized at the front of polarized cells and pseudopod protrusion (96). Since P2X1, P2X4, and P2X7 receptors are all expressed by CD4^+^ T cells, the authors identified the P2X receptor involved in T cell migration induced by CXCL12, using specific pharmacological inhibitors. They found that P2X4 inhibition by 5-BDBD has the strongest inhibitory effect on T cell migration, CD69 expression, and CD4^+^ T cell proliferation, while inhibition of P2X1 and P2X7 has a weaker effect on CD4^+^ T cells (96). Interestingly, after T cell stimulation, P2X4 receptors were concentrated at sites of pseudopod protrusion with activated mitochondria. Thus, an autocrine purinergic pathway modulates mitochondrial activity, which in turn activates P2X4 receptor leading to pseudopod formation and T cell migration.

In the continuation of their previous work, Ledderose et al. studied the role of the purinergic receptor P2Y11 in the migration of human T lymphocytes (97). The P2Y11 receptor is a G-protein coupled receptor which, after binding of extra-cellular ATP, induces the production of the second messenger c-AMP, which stimulates protein-kinase A. The stimulation of CXCR4 chemokine receptor by CXCL12 induces a quick release of ATP by T lymphocytes. Inhibition of P2Y11 by a selective pharmacological inhibitor or siRNA silencing blocked the migration of human CD4^+^ T cells or of Jurkat cells following CXCL12 treatment. Similarly, siRNA silencing of P2X4 or inhibition of P2X4 by 5-BDBD inhibited T cell migration induced by CXCL12. Thus, P2X4 and P2Y11 receptors are required for T cell migration induced by CXCL12 (97). In addition, P2Y11 receptors are localized at the posterior part of the cell, where they negatively modulate the activity of surrounding mitochondria. At the other pole of the T cell, mitochondrial ATP production amplifies P2X4 receptor activity (Ca^2+^ influx), which boosts mitochondrial ATP synthesis. The ATP production triggers actin cytoskeleton re-organization required for pseudopod protrusion and T cell migration. It is worth noting that mice do not express P2Y11 receptors (98). Thus, it would be interesting to determine whether another mouse P2Y receptor is able to functionally replace the human P2Y11 receptor and trigger c-AMP/protein-kinase A activity.

The studies described in the previous paragraphs were performed with T lymphocytes bearing the αβ TCR. Thus, Manohar et al. (99) determined whether purinergic signaling could amplify TCR responses of non-conventional T lymphocytes expressing γδ TCR. Using purified γδ T cells from human peripheral blood, they found that stimulation with anti-CD3/CD28 antibodies coupled to microbeads induced rapid release of ATP, reaching a maximal concentration 30 seconds after stimulation. They showed using pharmacological inhibitors that inhibition of pannexin-1 and connexin hemichannels blocked ATP release after TCR stimulation. In addition, inhibition of vesicular exocytosis with Brefeldin A significantly decreased ATP release, suggesting that this pathway is also involved in ATP release to the extracellular space. The authors also found that these lymphocytes express A_2A_, P2X1, P2X4, P2X7 and P2Y11 receptors (99). To determine which purinergic receptor may impact γδ T cell responses, they studied the expression of CD69, an early T cell activation marker, and the increase in TNF-α and IFN-γ mRNA transcripts following TCR stimulation after pharmacological inhibition of P2X1, P2X4, P2X7 and P2Y11 (99). They found that only P2X4 blockade inhibited TNF-α and IFN-γ transcription in response to γδ T cell stimulation with anti-CD3/CD28 coated beads (99). Thus, they concluded that ATP release and autocrine signaling *via* P2X4 receptors are involved in the modulation of γδ T cell responses.

Most of these studies were performed with human T lymphocytes or Jurkat cells activated principally by anti-CD3/CD28 antibodies coupled to microbeads or stimulated by CXCL12. It is important to determine whether similar purinergic signaling occurs in murine models. In particular, the physiological interactions between murine T lymphocytes and antigen presenting cells (APC) could be tested *in vitro* and *in vivo* using TCR–transgenic mouse lines specific for a defined antigen and another mouse line constitutively expressing the same antigen in APC. In addition, conditional knockout mice in which one can control the timing and tissue distribution of P2X4 receptor expression (available at EUCOMM/Genoway) could be used to delineate the role of P2X4 in physiological or pathological conditions at the immunological synapse. Thus, eliminating P2X4 expression (or other P2X receptors) from T lymphocytes or dendritic cells (professional APC) may shed light on their ability to amplify TCR responses, depending on their presence on T cells or APC.

## P2X4 and Function/Disease of Specific Organs

**Figure 3:**
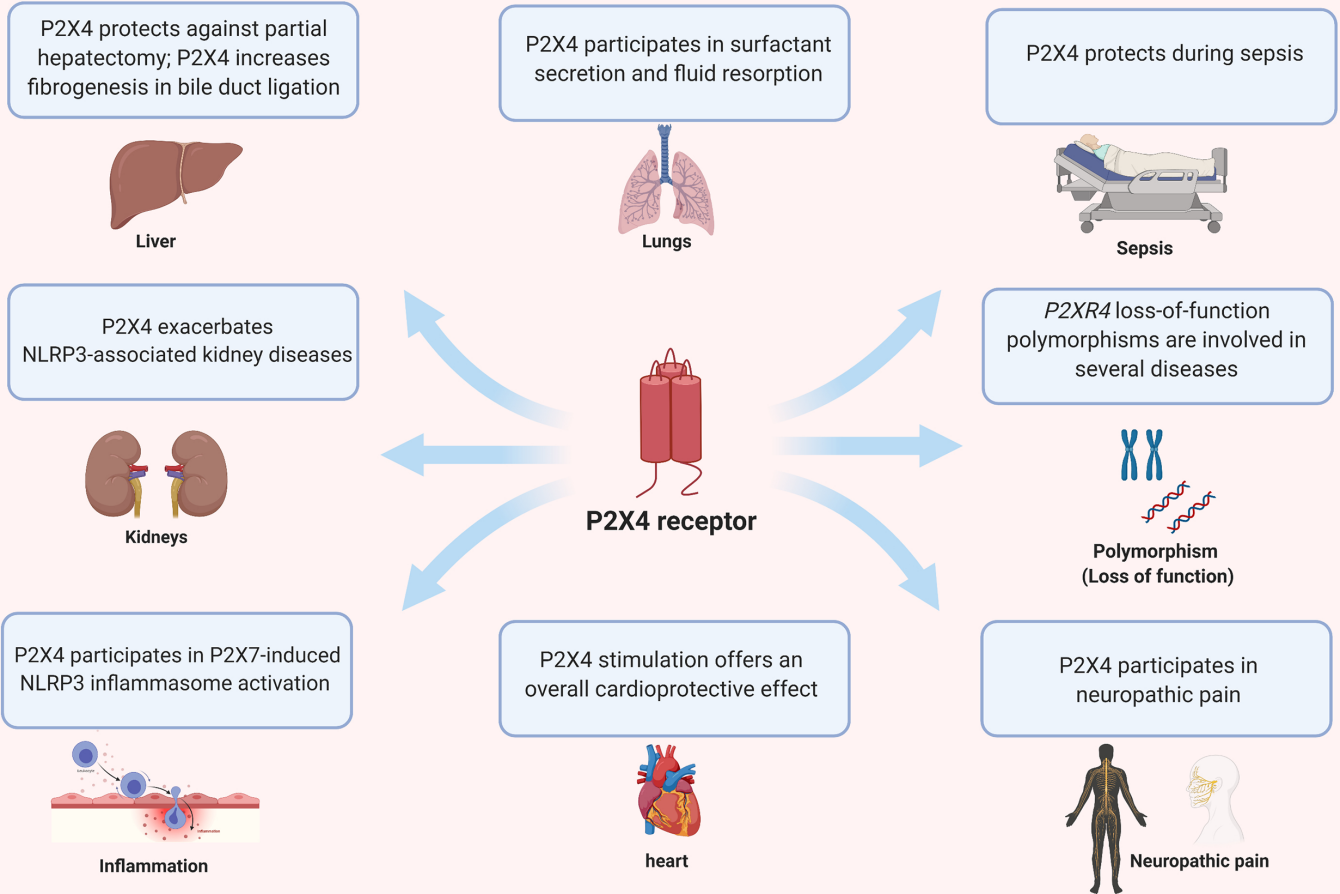
Pleiotropic effects of P2X4 on different organs and diseases.

P2X4 is expressed ubiquitously and modulates disease in various organs such as heart, kidney, liver, lung and CNS (**Figure 3**). In the following paragraphs, we review the role of P2X4 in the lungs, kidney, liver and auto-immune diseases of the nervous system. We refer readers to recent reviews on the role of P2 receptors in distal lungs for more details and informative illustrations (4), cardiovascular disease (48), neuropathic pain (47, 100) and CNS function and pathophysiology (101).

### P2X4 and Lungs

The P2X4 receptor has been described to be involved in lung function, specifically in vesicular release of lamellar bodies containing surfactant and surfactant release, control of fusion pore expansion and epithelial fluid resorption. Alveoli are found at the end of the bronchioles and are responsible for gas exchange. The alveolar epithelium comprises two main cell types: the alveolar type I (ATI) and alveolar type II (ATII) cells (102). ATI cells express P2X7 and P2Y2 receptors (4), cover >95% of the alveolar surface (103), and are responsible for efficient gas exchange between the alveolus and the pulmonary capillaries. ATII cells express P2X4, P2X7, P2Y2 and P2Y6 receptors (4). ATII cells synthesize, store and release pulmonary surfactant into the alveolar lumen, where they act to reduce the surface tension and optimize conditions for gas exchange (4, 102).

ATII cells contain lamellar bodies, which are large lysosome-related organelles with an acidic lumen that stores surfactants (104). Studies have shown that the P2X4 receptor is expressed on the surface of lamellar bodies in ATII cells, and is involved in the secretion and activation of surfactants (105, 106). Miklavc et al. reported that once lamellar bodies fuse with the plasma membrane to exert exocytosis, P2X4 receptors located in the vesicle promote fusion pore opening and exocytic content release in ATII cells (105). Unlike many readily-soluble molecules, surfactants are not immediately released following fusion of lamellar bodies with the plasma membrane (4). Once lamellar bodies fuse with the plasma membrane, P2X4 receptors located in the lamellar body are transiently activated and generate a localized cytosolic Ca^2+^ signal in the immediate vicinity of the fused lamellar body, which was termed as fusion-activated Ca^2+^ entry (FACE) (105). P2X4-induced FACE leads to an increase in intracellular Ca^2+^ at the fusion site, which is required for the fusion pore expansion. The expansion of the pore facilitates the release of the poorly soluble surfactant from the lamellar body to the alveolar lumen (4).

It was later demonstrated that the lamellar body itself carries all that is necessary (ion channel and its ligand) to promote pore expansion and surfactant release (107). Fois et al. showed that lamellar bodies store ATP, and that these molecules activate vesicular P2X4 receptor in an autocrine manner upon exocytosis, once the fusion pore is open in the plasma membrane of ATII cells (107). It is important to note that lamellar bodies have an acidic pH of approximately 5.5, and P2X4 receptors are strongly inhibited under low pH conditions (104). Thus, upon opening of the fusion pore, the intraluminar pH of lamellar bodies is neutralized, which then allows P2X4 receptors to be activated by ATP under neutral pH conditions. It has also been suggested that P2X4 activation and FACE are part of a positive feedback loop, in which the increase in Ca^2+^ concentrations near the cellular plasma membrane leads to additional lamellar body fusion (4, 108). These studies demonstrate the importance of the P2X4 receptor in the secretion of pulmonary surfactant and control of fusion pore expansion in ATII cells.

Efficient gas exchange and surfactant function depend on regulation of the alveolar surface liquid volume and composition (4). The activation of P2Y2 receptors by luminal nucleotides induce Cl^-^ secretion and inhibit Na^+^ absorption, leading to fluid secretion. On the other hand, FACE *via* P2X4 receptor promotes fluid resorption in response to surfactant secretion (4). It was described that P2X4 induces an inward cation current (via FACE), which in turn leads to apical-to-basolateral fluid transport (106). The localized alveolar fluid resorption temporarily decreases the level of alveolar surface liquid, which helps maintain an adequate environment for efficient gas exchange and lung function.

### P2X4 and Kidneys

Renal ischemia/reperfusion is a common etiology of acute kidney injury (AKI). It is a consequence of a defect in oxygen and nutrient supply to kidney cells, resulting in an accumulation of metabolic waste. This may lead to proximal tubular cell death by apoptosis/necrosis associated with inflammatory responses and infiltration of various leukocytes. Among DAMP molecules released by dead cells, ATP can ligate P2 receptors. P2X7 is barely expressed in normal kidney cells but can aggravate several kidney diseases such as acute kidney injury, diabetic nephropathy, septic AKI, and chronic kidney disease (109–112). However, P2X7 receptor expression increases during kidney inflammation, which can account for its effects in several kidney diseases. Surprisingly, while P2X4 is expressed in normal kidney cells (113), its role in kidney diseases has not been well defined yet.

As mentioned above in the section on inflammasomes and P2X4, in several kidney diseases when NALP3 inflammasome activation is involved in the physiopathology, P2X4 was found to exacerbate the disease. However, P2X4 does not always exacerbate pathology in kidneys. The role of P2X4 in unilateral ureteric obstruction (UUO), a rodent model of chronic interstitial inflammation and fibrosis, has been studied by Kim et al. (113). Higher accumulation of collagen fibrils, and higher expression of transforming growth factor-β and connective tissue growth factor mRNAs, was found in kidney tissues from UUO-operated P2X4-deficient mice, compared with wild-type control (113). In contrast, after UUO, comparison of UUO-operated kidneys showed a significant decrease in tubulo-interstitial damage characteristic of obstructive nephropathy in P2X7-deficient animals compared with wild-type animals (114). Thus, the lack of P2X4 results in significant enhancement of kidney tubulo-interstitial damage and fibrosis after UUO (113).

### P2X4 and Liver

Purinergic signaling in the liver in health and disease has been reviewed by Burnstock et al. (115). Different cell subsets in the liver express various P2 purinoreceptors of the P2Y and P2X families (115). P2X4 and P2X7 are well represented in various liver cell types, implying that these receptors may have important functions in liver physiology and/or physiopathology. It has previously been shown that partial hepatectomy (PH) in rats induces a rapid and short-lived ATP release from liver in blood and bile (116). This is due to mechanical stress following the strong increase in blood pressure in the liver and portal vein after elimination of two thirds of the hepatic blood vessels. ATP is released by Kupffer cells because removal of these cells by clodronate treatment correlates with a decrease of ATP in the hepatic vein (116). In addition, using quinacrine to stain ATP containing cell compartments, Gonzales et al. (116) showed that hepatocytes contain ATP-enriched lysosomes. After PH, contents of these ATP-containing lysosomes were released, probably by lysosome exocytosis (116). Importantly, ATP release correlates with hepatocyte cell cycle progression; and treatment of rats with PPADS, a pharmacological inhibitor of P2 purinoreceptors, inhibits liver regeneration (116). These experiments strongly suggest that P2X4 signaling may be involved in liver recovery after PH.

The same authors studied the role of P2X4 during acute and chronic liver injury (35, 117). They found that, in hepatocytes, P2X4 is expressed in ATP-containing lysosomes and that liver regeneration after PH is delayed in P2X4-deficient mice compared with wild-type animals. They showed that the absence of P2X4 does not alter hepatocyte proliferation but increases liver injury after PH, i.e. hepatocyte necrosis and cholestasis (35). After PH, P2X4-deficient mice show a decrease in bile flow with an increase of $HCO_{3}^{-}$ in bile, which is associated with a significant drop in biliary pH when compared with wild-type animals (35). In addition, modifications of lysosome biogenesis and early inflammatory reactions were found in P2X4-deficient mice after PH (35, 117).

In a model of liver chronic injury i.e. bile duct ligation (BDL), Le Guilcher et al. (117) studied the role of P2X4 in liver fibrogenesis associated with hepatic myofibroblasts responsible for the production of exaggerated extracellular matrix components. They showed that P2X4 is strongly expressed in hepatocyte myofibroblasts and controls myofibroblast contraction, adhesion and migration when BDL-induced fibrosis was compared in P2X4-deficient and wild-type mice (117). The pro-fibrogenic effects of P2X4 in BDL are due to lysosomal exocytosis from hepatocyte myofibroblasts, leading to the release of profibrogenic/profibrolytic components such as IL-6, PDGFb and VEGF-A, MMPs and TIMPs. Exocytosis of lysosomes also contribute to the release of ATP that had been stored in P2X4-expressing lysosomes. This may trigger P2X4-dependent activation of hepatocyte myofibroblasts. Thus, these results indicate that blocking P2X4 should inhibit fibrogenesis and may be considered as an anti-fibrosis strategy for treatment of chronic liver disease.

### P2X4 and Experimental Autoimmune Encephalomyelitis (EAE): An Animal Model of Multiple Sclerosis

ATP is highly concentrated inside cells under physiological conditions. It belongs to the family of DAMPs that are released by stressed cells or after cell lysis (118).

P2X4 is expressed at high levels in activated microglia of rats with EAE and in the optic nerves of multiple sclerosis (MS) patients (119). Thus, Zabala et al. (120) studied the role of P2X4 during myelin-oligodendrocyte glycoprotein (MOG)-induced EAE. They found that P2X4 is upregulated at the peak of EAE and persisted during the recovery phase. In addition, they observed that Interferon regulatory factor (Irf)-8 and Irf-5 transcription factors (121, 122) were upregulated at the peak and recovery phases of EAE, and that their levels were consistent with the expression level of P2X4. On the basis of their previous work (119), they hypothesized that P2X4 inhibition should prevent microglial death and recruitment of inflammatory monocytes at early stages of EAE induction. Nonetheless, in apparent disagreement with their hypothesis, blockade of P2X4 by daily injection of the P2X4 antagonist TNP-ATP exacerbated EAE disease (120). Importantly, they showed that EAE severity was increased in P2X4-deficient mice and that TNP-ATP treatment did not modify the course of the disease in P2X4-deficient animals. As myelin clearance is necessary for remyelination and phagocytosis/remyelination is dependent on microglia polarization, the authors compared gene expression in the lumbar spinal cord of vehicle- and TNP-ATP injected mice at the peak and recovery phase of EAE. They found that inhibition of P2X4 only increased the pro-inflammatory genes during EAE and induced pro-inflammatory polarization of microglia, leading to a decrease in myelin phagocytosis by microglia and low remyelination of oligodendrocytes. Importantly, Zabala et al. (120) used ivermectin, a positive potentiator of P2X4, to evaluate its therapeutic potential in EAE. When this drug was administered daily after the onset of EAE, a significant improvement of the motor deficits and of the axon conduction latencies in the corticospinal tract were observed. The effect of ivermectin on *in vitro* microglial polarization showed that pro-inflammatory genes decreased while anti-inflammatory genes increased. In addition, ivermectin triggered endosome-lysosome fusion and increased phagocytic activity, potentiating myelin degradation and stimulating remyelination. The action of ivermectin was P2X4-specific because it was not observed in P2X4-deficient microglia. These results suggest that ivermectin, a drug already approved for use in human parasitic diseases, should be tested for treatment of MS.

## P2X4 as a Mediator of Ethanol Action

P2X4 ligand-gated ion channels are the most highly-expressed and most ethanol-sensitive P2X receptors in the central nervous system (123) and also mediate the main effect of ethanol on the brain (124). Alcohol increases P2X4 expression on the cell surface (125) and, at the same time, inhibits signaling *via* P2X4 receptors (126). P2X4 activity is selectively blocked by ethanol, which acts as a negative allosteric modulator that blocks the open channel (123, 127).

P2X2 and P2X4 expressed in *Xenopus* oocytes can inhibit ATP-associated currents at toxic and anesthetic concentrations of ethanol (127). However, ethanol inhibition does not depend on either voltage or ATP concentration but may alter the receptor affinity for ATP (123). Using the whole-cell patch-clamp technique in HEK293 cells, ethanol was found to inhibit P2X4 channel opening by affecting receptor function at amino acid residues Asp331 and Met336 in the ectodomain of transmembrane region 2 (TM2). The results also showed that ethanol inhibits P2X4 channel function through a mechanism different from that of other P2XRs (123). Modeling data further suggest that these residues may be part of the alcohol interaction pocket (128). However, it is not possible to distinguish between ethanol interacting directly with Asp331/Met336 residues and ethanol interacting with separate loci that alter the channel conformation at these positions, making the channel unable to bind ethanol (123).

It is noteworthy that these same amino acids also play a role in the interaction of ivermectin with P2X4. Ivermectin can antagonize the inhibitory effect of ethanol on P2X4 and is thought to interfere with ethanol binding to the channel (128). Trp46 in the TM1 segment of the receptor has also been shown to contribute to the formation of a putative binding pocket (129). Recent studies confirm that ethanol has several binding sites on the P2X4 receptor - high affinity binding sites and low affinity binding sites. First, high-affinity sites (such as position Met336) that are sensitive to the lower intoxicating concentrations experienced by most social drinkers, and second, low-affinity sites (such as positions 46 and 331) that show responses to higher alcohol concentrations achieved by alcoholics. Taken together, these results suggest that there is a direct interaction between ethanol and P2X4, which may have a significant effect on alcohol use disorders (126).

The results of a genomic study suggest that P2X4 may play a role in alcohol consumption and preference in mice and rats. Rodents lacking the *P2RX4* gene drink more ethanol than wild-type counterparts (126). In particular, whole brain expression of the *P2RX4* gene is inversely related to alcohol preference and P2X4 mRNA expression is reduced in alcohol-preferring rats (130).

Interestingly, purinergic receptors, such as P2X4, can affect satisfying and repulsive neural chains – in particular, those involving dopamine, thereby involving these receptors in modulating the addictive synaptic signal. Therefore, P2X4 may be an important therapeutic target in the treatment of alcohol abuse behavior (128). P2X4 activity has also been implicated in other behaviors associated with dopaminergic neurotransmission, including motor control and the handling of sensory information (131).

Alcohol is known to cause structural and functional disorders in microglial cells, which include phagocytosis, cell proliferation, and migration. For example, P2X4 is significantly upregulated in microglial cells after exposure to ethanol. Interestingly, after ethanol treatment, both microglial phagocytosis and migration toward CX3CL1 decrease in a P2X4-dependent fashion (125). A recent publication reports that an ethanol-sensitive component of ATP is mediated by P2X4, since the ventral tegmental area neurons are inhibited by ATP, and ethanol antagonizes this inhibition (132). Taken together, these findings suggest that alcohol consumption may be modulated through P2X4, and that ivermectin or ivermectin analogs have the pharmacological potential to reduce alcohol consumption and preferences (5).

## Polymorphisms of Human P2X4

Genetic polymorphisms may lead to a gene sequence variation that is neutral, or promotes gain- or loss-of-protein function. Some genetic variations can affect clinical conditions in which relevant proteins are slightly modified, leading to a decrease or increase in disease susceptibility. The *P2RX7* gene is highly polymorphic in humans, showing at least eight non-synonymous single nucleotide polymorphisms (SNPs) that can lead to a functional effect on the P2X7 receptor (133). The human *P2RX4* gene is located on chromosome 12q24.32 (134) and has 12 exons. The human *P2RX4* gene may present four non-synonymous SNPs, in which three of them have no significant effects on the receptor, but one leads to a loss-of-function P2X4 receptor (135).

The loss-of-function polymorphism (Tyr315Cys) in the *P2RX4* gene was associated with increased susceptibility to age-related macular degeneration (AMD) in a study of 744 patients and 557 age-matched Caucasians (136). Interestingly, haplotype analysis showed that the P2X4 315-Cys minor allele co-inherited with P2X7 150-Arg was present 4-fold more often in patients with AMD than control subjects. Reduced phagocytic activity was observed in cells expressing both P2X4 315-Cys and P2X7 150-Arg variants, which led the authors to speculate that a decrease in macrophages/microglia phagocytic functions, and therefore a decrease in debris clearance, may contribute with the onset of AMD (136).

The P2X4 receptor is abundantly expressed in endothelial cells and is required for optimal flow-sensitive mechanisms that regulate blood pressure and vascular remodeling (137). Additionally, P2X4-deficient mice present reduced endothelial cell responses to blood flow and develop hypertension (137), suggesting that a loss-of-function polymorphism in the *P2XR4* gene in humans could affect blood pressure. In fact, one study showed that genetic variations in P2X4 (and also P2X7) are linked with a small but significant effect on blood pressure in a Caucasian population (138). A different group reported that a loss-of-function polymorphism in the P2X4 receptor (Tyr315>Cys, the same mutation described in the study regarding AMD) significantly reduced ATP-induced inward currents at the cellular level, which is likely due to alterations in the agonist binding site of the receptor (135). Analysis of this genetic variant in 2874 subjects demonstrated that the Tyr315>Cys mutation was significantly associated with pulse pressure. The increase in pulse pressure may be a reflection of P2X4 loss-of-function conferred by the Tyr315>Cys mutation (135). In fact, using human umbilical vein endothelial cells (HUVECs), a separate study demonstrated that kruppel-like factor 2 (KLF2) is involved in shear stress regulation through P2X4 activation. Transfection of *P2RX4* loss-of-function genetic variant Tyr315>Cys into human endothelial cells blocked ATP-induced KLF2 expression. These studies suggest an important role for P2X4 in endothelial cell-modulated blood pressure regulation.

Analysis of SNP polymorphisms in the *P2RX4* gene suggested an association of this gene with susceptibility to HIV-associated sensory neuropathy (139), even though there was a stronger association of the disease with the P2X4 contiguous gene, CAMKK2. The authors suggested that polymorphisms in the *P2RX4* gene and association with HIV-associated sensory neuropathy may be due to the role of P2X4 in pain (139) and the fact that some patients presenting specific *P2RX4* alleles had increased TNF-α concentrations in their PBMC (140), which contributes to the onset of disease.

The fact that P2X4 is expressed in both osteoclasts and osteoblasts suggests a role for this receptor in bone physiology. A study evaluated patients with fractures (690 females and 231 males) and found that subjects carrying the loss-of-function Tyr315Cys genetic variant presented decreased bone mineral density values and showed 2.68-fold higher risk of osteoporosis compared with controls (141). The same group previously showed that a P2X7 SNP is associated with decreased bone mineral density values (141). However, whether P2X4 and P2X7 SNPs synergistically contribute to increase the risk of osteoporosis is still unclear.

*P2RX7* variants resulting in loss-of-function were found to be protective against multiple sclerosis, whereas variants conferring gain-of-function increased the risk of disease (142). A separate study evaluated 197 multiple sclerosis patients and 100 control subjects and identified a *P2RX7-P2RX4* haplotype containing three rare missense mutations associated with high incidence of multiple sclerosis (143). Sadovnick et al. (143) identified 3 variants (P2X7 T205M, P2X7N361S, P2X4 G135S) that co-segregate with MS in one family where 6 individuals out of 13 were affected by the disease. When expressed in HEK293 cells, they found that P2X7 T205M had lost its plasma membrane expression and phagocytic activity (143). In contrast, the function of P2X7 N361S was undistinguishable from WT P2X7. The P2X4 G135S mutant expressed in HEK293 cells was associated with an increase in ATP-induced inward currents and a higher response to Ca^2+^, compared with wild-type P2X4 (143). Thus, the P2X7 T205M variant has a strong impact on P2X7 function, while the two other mutations have a minor effect on P2X4-P2X7. Additional studies are required to determine whether these two rare variants (P2X7 N361S, P2X4 G135S) are also risk factors in MS in the absence of P2X7 T205M.

Altogether, studies from this section suggest that subjects carrying genetic variants of the *P2RX4* gene may have higher risks for developing HIV-associated sensory neuropathy, blood pressure complications, osteoporosis, multiple sclerosis, and impaired immune responses. Since most of the studies were conducted in a Caucasian cohort population for a limited number of diseases, future studies will evaluate the role of *P2RX4* gene variants in different races, and whether the *P2RX4* gene plays a role in other pathological conditions.

## Conclusion

Most studies on purinergic function until now have focused primarily on P2X7. Less is known about the function of P2X4 under normal conditions and during pathogenesis. Comparisons between the two receptors is facilitating the structural characterization of P2X4. Nonetheless, the contribution of P2X4 to disease states appears to be mostly different from the role of P2X7, and the tissue distribution for the two receptors is different. Polymorphisms in the P2RX4 gene has led to discoveries about the possible function of P2X4 in human physiology. Little is known about the role of P2X4 in modulating inflammation and in immune responses to infection. Further studies in the future should shed light on the function of this understudied purinergic receptor.

## Author Contributions

JK, CA-D, SRB, and DO have each written different sections of the manuscript. CA-D made the figure. All authors contributed to the article and approved the submitted version.

## Funding

This study was partially supported by funds provided from The Regents of the University of California, Tobacco-Related Diseases Research Program, grant T29FT0540 to CA-D. The opinions, findings, and conclusions herein are those of the authors and not necessarily represent those of The Regents of the University of California or any of its programs. JK was supported by the Institut national du cancer (INCa) project P2X7R 2015-137. SRB was supported by the Estonian Research Council grant COVSG34. **Figure 3** was created in BioRender.com. We warmly thank Dr. Pierre Boudinot for his suggestions and help with the drawing of our figures.

## Conflict of Interest

The authors declare that the research was conducted in the absence of any commercial or financial relationships that could be construed as a potential conflict of interest.
